# Efficient Non-Uniform Pilot Design for TDCS

**DOI:** 10.3390/s21206880

**Published:** 2021-10-17

**Authors:** Cheng Chang, Lina Feng, Hui Zhou, Zilong Zhao, Xin Gu

**Affiliations:** 1 China Academy of Launch Vehicle Technology, Beijing 100076, China; bhzhouhui@163.com (H.Z.); zhaozilong1214@126.com (Z.Z.); guxincalt@126.com (X.G.); 2 Beijing Institute of Astronautical Systems Engineering, Beijing 100076, China; flnhit@126.com

**Keywords:** Internet of Things, transform domain communication system, non-continuous spectrum, fast-varying channel, non-uniform pilot, efficiency

## Abstract

The Internet of Things (IoT) leads the era of interconnection, where numerous sensors and devices are being introduced and interconnected. To support such an amount of data traffic, wireless communication technologies have to overcome available spectrum shortage and complex fading channels. The transform domain communication system (TDCS) is a cognitive anti-interference communication system with a low probability of detection and dynamic spectrum sensing and accessing. However, the non-continuous and asymmetric spectrum brings new challenges to the traditional TDCS block-type pilot, which uses a series of discrete symbols in the time domain as pilots. Low efficiency and poor adaptability in fast-varying channels are the main drawbacks for the block-type pilot in TDCS. In this study, a frequency domain non-uniform pilot design method was proposed with intersecting, skewing, and edging of three typical non-uniform pilots. Some numerical examples are also presented with multipath model COST207RAx4 to verify the proposed methods in the bit error ratio and the mean square error. Compared with traditional block-type pilot, the proposed method can adapt to the fast-varying channels, as well as the non-continuous and asymmetric spectrum conditions with much higher efficiency.

## 1. Introduction

The Internet of Things (IoT) is a global network of interconnected objects, where numerous sensors and devices are interconnected as parts of the internet to expand their efficiency [1]. Wired and wireless networks are ubiquitous in IoT, which largely increases the demand on the spectrum overhead [2,3]. Cognitive radio (CR) is an emerging trend for supporting multiuser and hybrid communications [4,5]. With the combination of IoT and CR concepts, the network is applicable for real-time cognitive radio applications as well as various dynamic environments [6,7,8,9]. Transform domain communication system (TDCS) is a cognitive anti-interference communication system, which is regarded as a promising candidate of CR for the IoT massive multiple access scenarios [10,11]. Unlike orthogonal frequency division multiplexing (OFDM) and multi-carrier code division multiple access (CDMA), TDCS is designed to avoid occupied frequency bins by signal processing facilities at both the transmitter and receiver instead of mitigating the interference only at the receiver [12,13,14]. Therefore, sensing the spectrum at both sides and shaping the transmitting waveform are the key features of TDCS.

In practical applications, TDCS has to adapt to the multipath time-varying channels. Without accurate channel state information (CSI), TDCS either cannot work or may suffer serious performance losses. Besides, the non-continuous and asymmetric spectrum of the transmitter and the receiver make the channel estimation and equalization more difficult. Pilots are a common solution for obtaining the CSI [15]. For TDCS, only block-type pilots are reported in the literature, which periodically inserts certain symbols in the time domain of the transmitting signal. Since pilots appear in all unoccupied frequency bins of the inserted symbols, the block-type pilots are insensitive to the frequency selective fading. In other words, they are only valid for the slow-varying channels, where the CSI is constant during one block [16,17]. Besides, the pilot symbols could not carry data, which reduces the system efficiency. Comb-type pilots are widely used in OFDM [18]. They uniformly distribute in the frequency domain of every symbol, which leads to a better flexibility for the fast-varying channels. Since the CSI is obtained after frequency domain interpolation, comb type pilots are sensitive to the frequency selective fading [19]. However, in TDCS, uniform pilots are not suitable for the non-continuous spectrum conditions [20]. Besides, some convex optimization methods based on non-continuous OFDM are seriously affected by the asymmetric spectrum between the transmitter and the receiver [21]. In this article, a series of efficient non-uniform pilots are proposed in the frequency domain in the forms of intersecting, skewing, and edging types. Compared with the existing pilot types, the proposed methods are more adaptive to the fast-varying channels, as well as the non-continuous and asymmetric spectrum conditions with much higher efficiency.

In Section 2, TDCS and the classification of the typical transmitters and receivers are reviewed as the foundation of the subsequent studies. In Section 3, efficient non-uniform pilots are designed for TDCS, whose performance is also analyzed with comparisons. In Section 4, some numerical examples are presented to verify the proposed methods. The article is then concluded in Section 5.

## 2. TDCS and the Classification of the Transceivers

### 2.1. TDCS Model

**Figure 1:**
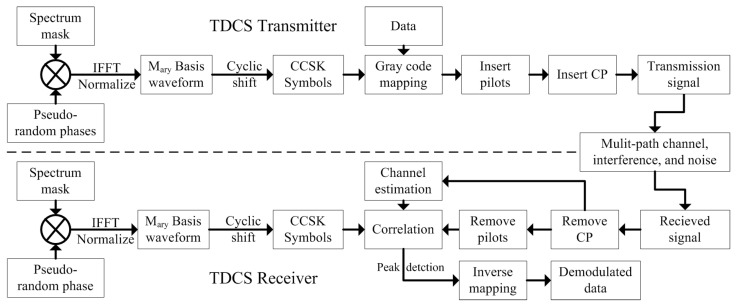
The diagram of TDCS.

The TDCS model is depicted in Figure 1. The transmitter and the receiver independently sense the whole bandwidth to create the spectrum mask A(k), with the value 1 or 0 if the kth frequency bin is unoccupied or interfered. Pseudo-random phases $\theta_{k}$ are created by a pseudo-random sequence generator and applied element by the element to the spectrum mask. The resulting vector is then passed through an inverse fast Fourier transform (IFFT) and scaled to the desired power. Then, the basis waveform is cyclic-shifted to modulate data in Gray code. The ith modulated symbol in the frequency and the time domain can be expressed in complex baseband notation as (1) and (2), respectively.

(1) $$S_{TDCS,i}(k)=\sqrt{\frac{N}{N_{1}}}A(k)e^{j\theta_{k}}e^{-j2\pi m_{i}k/M}$$

(2) $$s_{TDCS,i}(n)=\frac{1}{\sqrt{NN_{1}}}\sum_{k=0}^{N-1}A(k)e^{j\theta_{k}}e^{-j2\pi m_{i}k/M}e^{j2\pi kn/N}$$

In the equations above, N and $N_{1}$ are the numbers of the total and the unoccupied frequency bins, respectively. $m_{i}\in [1,M]$ is the ith data with M-ary CCSK (cyclic code shift keying) modulation [22].

In the transmitter of TDCS, pilot symbols are then periodically inserted to the modulated symbols in the time domain. After inserting the cycle prefix (CP) to every symbol [23], the transmitting signal is completely generated.

The transmitting signal goes through the multi-path channels with interferences and additive white Gaussian noise (AWGN). In the receiver, the CP is removed from the received signal, the pilots are extracted to estimate the CSI. The rest are the data symbols, which are used for correlation and peak detection with the estimated CSI. Finally, the demodulated data can be obtained by the corresponding inverse mapping.

### 2.2. The Classification of TDCS Transmitters and Receivers

Considering the influence from predetermined conditions of the frequency domain pilot interval and the practical spectrum mask generation, we can classify TDCS transmitters into two categories:

1. Tx 1;

The two factors above act independently. The pilots are uniformly inserted in the frequency domain of every symbol, which are designed the same as the contiguous spectrum. The practical spectrum mask restricts the interfered frequency bins for transmission [24].

2. Tx 2;

The practical spectrum mask can influence the frequency domain pilot interval. Exhaustion or convex optimization methods insert pilots non-uniformly in the unoccupied frequency bins.

According to whether the receiver knows the real-time pilots of the transmitter, the receivers can be classified as:

1. Rx 1;

The receiver knows the transmitter practical spectrum mask or the positions of the pilots. Some spectrum exchange mechanisms were implemented to obtain the accurate pilots for channel estimation and interpolation. If the pilot design method is known, pilots on the symmetric frequency bins of both Tx 1 and Tx 2 type can be obtained.

2. Rx 2;

The receiver does not know the transmitter practical spectrum mask. This means there is no spectrum exchange between the transmitter and the receiver. If the spectrum is symmetric, the receiver can obtain the accurate pilot positions. For asymmetric spectrum conditions, partial pilots on the asymmetric frequency bins of Tx 1 type would be influenced. However, almost all pilots of Tx 2 type are influenced since different spectrum masks lead to entirely different pilot positions, and orders are confused [25].

Actually, the transmitter and the receiver of TDCS usually work in the non-continuous and asymmetric spectrum conditions. The spectrum exchange mechanism would occupy too much control signaling spending [26]. Therefore, to ensure Rx-2-type receivers work properly, the transmitter should be modified based on the Tx 1 type.

## 3. Efficient Non-Uniform Pilot Design for TDCS

### 3.1. Non-Uniform Pilot Design

For uniform pilots, according to the Nyquist sampling theorem [27], to restore frequency domain signal without distortion, the corresponding time domain extension period should be less than the maximum delay spread.

(3) $$N_{f}\leq \frac{1}{\tau_{\max}\Delta f}$$

**Figure 2:**
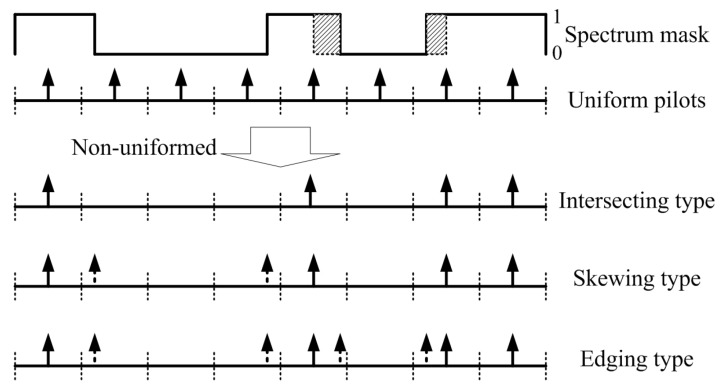
Non-uniform pilot design for TDCS.

In (3), $N_{f}$ is the minimum pilot interval in the frequency domain, Δf is the interval between adjacent frequency bins, and $\tau_{\max}$ is the maximum multipath delay. As shown in Figure 2, the spectrum of the whole bandwidth is divided into $N_{s}$ segments (separated with the dotted lines), and the uniform pilots lie in the center of every segment.

To fit the non-continuous and asymmetric spectrum conditions, we designed non-uniform pilots gradually. The simplest method is to directly use the intersecting between the uniform pilots and the practical spectrum mask. The positions of intersecting type $P_{\mathrm{int}}(k)$ can be designed as the uniform type pilot.

(4) $$P_{\mathrm{int}}(k)=P_{uniform}(k)A(k)$$

$P_{uniform}(k)$ is the uniform pilot mask with a value 1 or 0 if the kth position is pilot or not. In the positions of $P_{\mathrm{int}}(k)=1$, the values of the pilots $S_{\mathrm{int}}(k)$ can be designed as the absolute values of the frequency domain basis waveform.

(5) $$S_{\mathrm{int}}(k)=\left\{ \begin{array}{l} \left|S_{TDCS,i}(k)\right|,\;pilot\;positions\\ S_{TDCS,i}(k),else \end{array}\right.$$

The intersecting type is restricted by the practical spectrum mask. However, some segments are usually partly unoccupied with the uniform pilot positions interfered. This means the segments carry information, but no corresponding pilots are inserted to the segments. Therefore, the CSI of those segments could not be estimated, and their carried information may not be correctly demodulated. To solve the problem above, we designed skewing-type non-uniform pilots by appropriately skewing the pilots in the segments.

The skewing type was designed based on the intersecting type, if the uniform pilot position is unoccupied or the whole segment is interfered, which was set the same as the intersecting type. If the segment is partly unoccupied with the uniform pilot position interfered, the pilot position should skew using the following rule.

(6) $$O=[-1,+1,-2,+2\ldots \ldots -\frac{N/N_{s}-1}{2},+\frac{N/N_{s}-1}{2}]$$

In (6), O is the value of the skewing and − and + mean skewing in descending and ascending order within the segment, respectively. The values of the pilots are the same as the intersecting type in (5). As shown in Figure 2, the extra dotted arrowed pilots in the skewing type ensure the partly interfered segments can be estimated. Therefore, the most applicative spectrum conditions for the skewing type are dispersive and dense to ensure every segment has a valid pilot.

We assumed that the availability of each frequency bin within the total bandwidth N follows a binomial distribution B(N,p), where p is the probability of the availability. If only one pilot is inserted in each segment, the probability of the none frequency bin is available within a continuous s frequency bins, which could be deduced.

(7) $$P(s,p)={\left(1-p\right)}^{s}$$

According to (7), for the typical case with p=0.5 and s=8, the probability could be easily calculated as P(8,0.5)=0.0039, which would hardly happen. In other words, a valid pilot would be correctly inserted into every segment to achieve the channel estimation in the vast majority of cases. However, in actual fact, compared with multi-tone interference occupying on different frequency bins, narrowband or wideband interference are more common [28]. Therefore, to obtain a compete channel response, the restored frequency domain pilots must be interpolated, with frequently used constant interpolation, Gaussian interpolation, or cubic spline interpolation [29].

If the spectrum conditions are aggregate and sparse, the edges of the segments could not be correctly interpolated and estimated. In Figure 2, the shaded parts of the spectrum mask could not be interpolated for the non-valid pilot between them. We designed the edging type pilot based on the skewing type. Extra pilots were inserted in the edges of every interference (continuous zeros in the spectrum mask) with the vales as (5). In Figure 2, the dotted arrowed pilots in the edging type are the extra pilots.

### 3.2. System Design with Non-Uniform Interpolation, Estimation, and Equalization

**Figure 3:**
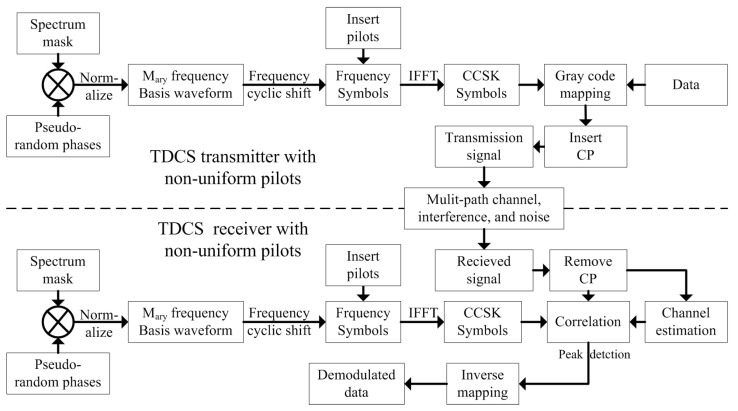
TDCS with non-uniform pilots.

TDCS with frequency domain non-uniform pilots in Figure 3 is quite different with a time domain pilot system in Figure 1. To avoid the extra process in the time domain, we directly generated M-ary frequency symbols in the frequency domain by multiplying spectrum mask, pseudo-random phases, and CCSK phases 2πmk/M.

(8) $$S_{m}(k)=\sqrt{\frac{N}{N_{1}}}A(k)e^{j\theta_{k}}e^{j2\pi mk/M}$$

In (8), m∈[1,M] is the sequence of M-ary CCSK. Then, the pilots were inserted in different types mentioned above with the absolute values of $S_{m}(k)$ in the pilot positions to generate CCSK symbols $S_{m,pilot}(k)$.

(9) $$S_{m,pilot}(k)=\left\{ \begin{array}{l} \left|S_{m}(k)\right|,pilot\;positions\\ S_{m}(k),else \end{array}\right.$$

The ith signal with data $m_{i}$ can be expressed.

(10) $$s_{i}(n)=\frac{1}{\sqrt{NN_{1}}}\sum_{k=0}^{N-1}S_{m_{i},pilot}(k)e^{j2\pi kn/N}$$

To eliminate the inter-symbol interference (ISI), the length of CP follows the rule as (11) [30].

(11) $$L_{CP}\geq \frac{\tau_{\max}}{t_{s}}=\tau_{\max}f_{s}$$

In (11), $\tau_{\max}$ is the maximum multi-path delay and $f_{s}$ and $t_{s}$ are the sampling frequency and sampling period, respectively.

In the receiver, the removed CP signal $r_{i}(n)$ was used for demodulation. According to the non-uniform pilot positions, we extracted pilots $P_{i}(k)$ from $R_{i}(k)$, which is the FFT of $r_{i}(n)$.

(12) $$R_{i}(k)=\frac{1}{N}\sum_{k=0}^{N-1}r_{i}(n)e^{j2\pi kn/N}$$

We used the least squares (LS) method [31] to estimate the channel response in the frequency domain.

(13) $${\hat{H}}_{i}(k)=\frac{P_{i}(k)}{P_{i,local}(k)}$$

In (13), ${\hat{H}}_{i}(k)$ was only valid in the pilot positions, while others were set as zero. $P_{i,local}(k)$ is the local accurate pilot. ${\hat{H}}_{i}(k)$ was non-uniform, and interpolation was necessary to fill the whole channel response according to the existing pilots. Uniform linear interpolation is first-order and fits the small pilot interval conditions. Uniform Gaussian and cubic spline interpolations are high-order, which leads to the variance of noise being doubled.

In this study, we regarded uniform linear interpolations between every adjacent non-uniform pilots as non-uniform linear interpolation to obtain the whole channel response ${\hat{H}}_{i,\mathrm{intp}}(k)$.

(14) $${\hat{H}}_{i,\mathrm{intp}}(x)=(1-\frac{q-x}{p-q-1}){\hat{H}}_{i}(q)+(1-\frac{x-p}{p-q-1}){\hat{H}}_{i}(p)$$

In (14), p and q were the adjacent pilots. x∈(p,q) are the interpolated positions. Minimum mean square error (MMSE) equalization [32] was used to eliminate the multi-path influence. The signal after equalization $r_{i,MMSE}(n)$ could be deduced.

(15) $$r_{i,MMSE}(n)=\frac{1}{\sqrt{NN_{1}}}\sum_{k=0}^{N-1}R_{i}(k)F_{i}(k)e^{j2\pi kn/N}$$

(16) $$F_{i}(k)=\frac{C({\hat{H}}_{i,\mathrm{intp}}(k))}{{\hat{H}}_{i,\mathrm{intp}}(k)C({\hat{H}}_{i,\mathrm{intp}}(k))+\sigma_{n}^{2}I_{1\times N}}$$

In the two equations above, $\sigma_{n}^{2}$ is the variance of the AWGN, $I_{1\times N}$ is the all-ones matrix with one row and N columns. C(x) means the conjugate of a complex matrix x.

To keep the signal-to-noise ratio (SNR) in the same level, we demodulated with pilots by using $s_{m}(n)$ (the time domain CCSK symbols with pilots) in the receiver to correlate with $r_{i,MMSE}(n)$. The peak detection and inverse mapping were the same as traditional TDCS in Figure 1.

### 3.3. Performance Analysis

For the time-domain block-type pilots, the pilots themselves occupied the symbols that are used for carrying information. Similar to (3), the sampling rate $1/N_{t}T$ should be not less than twice the signal bandwidth. The intervals between symbols can be expressed as (17). The efficiency $N_{t}$ can be defined as (18), which declines with the number of pilots.

(17) $$N_{t}\leq \frac{1}{2f_{d}T}$$

In (17), $f_{d}$ is the Doppler frequency, and T is the symbol duration. $N_{t}$ is the time domain interval according to the Nyquist sampling theorem.

(18) $$\eta_{t}=\frac{N_{t}}{N_{t}+1}$$

In TDCS, the CCSK constellation size M should meet the dimensionality theorem to ensure orthogonality [33].

(19) $$M\leq N_{1}$$

For frequency domain pilots, the pilots themselves occupy only some frequency bins rather than whole symbols. However, the pilots still occupy the power of those frequency bins, which are used for carrying information. The efficiency $\eta_{f}$ can be defined as Equation (20). Especially, based on (19), if it satisfies $M+N_{p}\leq N_{1}$, the efficiency of the TDCS with frequency domain non-uniform pilots can be constant $\eta_{f}=1$.

(20) $$\eta_{f}=\left\{ \begin{array}{l} \frac{N_{1}-N_{p}}{N_{1}},\;M+N_{p}>N_{1}\;\\ 1,\;M+N_{p}\leq N_{1} \end{array}\right.$$

In (20), $N_{p}$ is the number of the non-uniform pilots. M frequency bins ensure the CCSK orthogonality, while other $N_{p}$ frequency bins were used as pilots. The $N_{p}$ pilots just occupied the surplus dimensions.

The pilots were contained in every symbol, which made frequency domain non-uniform pilots fit to the fast-varying channels. The mean square error (MSE) can be expressed as (21).

(21) $$MSE({\hat{H}}_{i,\mathrm{intp}})=E\{{\left\|\left({\hat{H}}_{i,\mathrm{intp}}(k)-H_{i}(k))A(k\right)\right\|}^{2}\}$$

In (21), $H_{i}$ is the true channel response with pilots in all unoccupied frequency bins. E{⋅} is the mean of all unoccupied frequency bins.

Compared with convex optimization methods [34,35], the proposed methods ensure that the symmetric part of the frequency bins could be correctly estimated and equalized. The asymmetric part cannot be accumulated to the matching peak of the correlated demodulation in the receiver. Therefore, the SNR loss only appeared in the asymmetric parts.

## 4. Numerical Simulations

In this section, some numerical examples are simulated to verify the proposed methods. The parameters were set as bandwidth B=60 MHz, number of frequency bins N=256, and multipath model COST207RAx4 [36,37]. The bandwidth of every frequency bin was 234 KHz, while other signals and interferences in the L band could be regarded as large frequency blocks. Therefore, the spectrum mask was composed of interfered and unoccupied frequency blocks.

**Figure 4:**
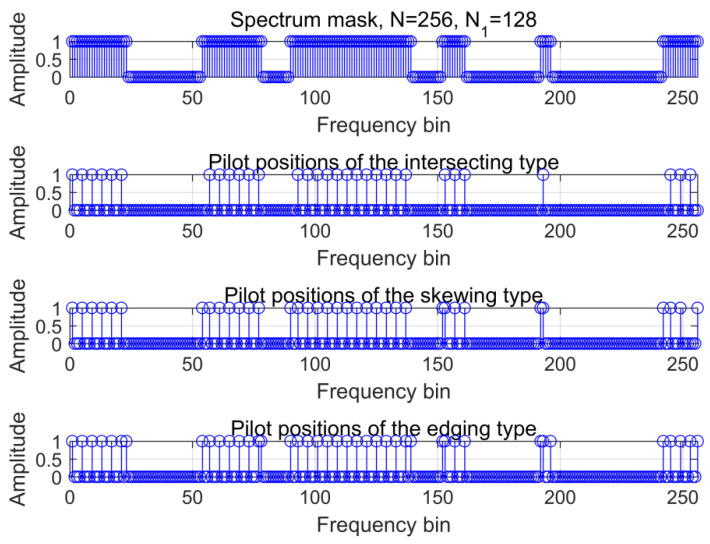
The non-uniform pilot positions within the bandwidth.

According to (5) and (6), the non-uniform pilot positions of the intersecting, the skewing, and the edging types are located as Figure 4 with $N_{1}=128$ and uniform pilot initial interval two frequency bins. Corresponding to the spectrum mask in the first row, the pilots’ positions are listed with in the bandwidth. To exactly restore the TDCS signal parts in the unoccupied frequency blocks as shown in the spectrum mask, the numbers of the skewing- and edging-type pilots were slightly greater than the intersecting type with an extra five and seven pilots, respectively.

**Figure 5:**
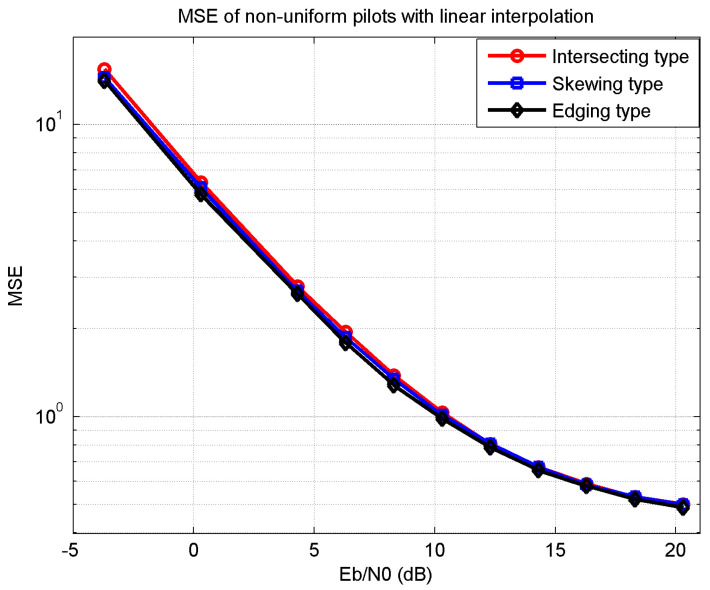
MSE of non-uniform pilots with linear interpolation.

Figure 5 shows the MSE of the three proposed pilot types with linear interpolation. The MSE decreases with SNR $E_{b}/N_{0}$,which implies that AWGN enlarges the difference between the estimated and the actual channel response. The number of the practical inserted pilots was slightly different, as in Figure 4, which led to that channel’s estimation performance of the edging-type pilot being slightly better than the others.

**Figure 6:**
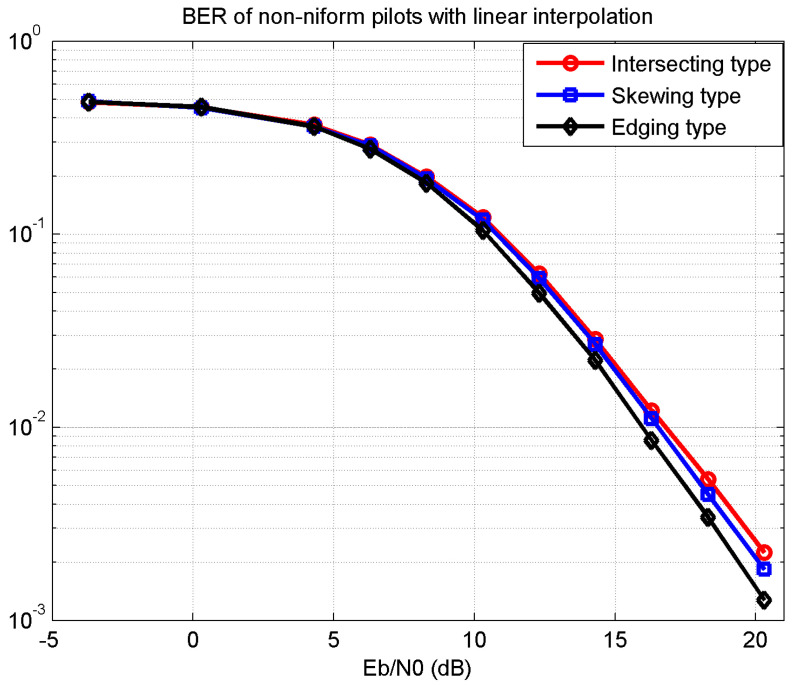
BER of non-uniform pilots with linear interpolation.

Figure 6 shows the BER performance of the three proposed pilot types with linear interpolation. The channel estimation of the edging type pilot was more accurate than the others; therefore, it needed a much lower SNR $E_{b}/N_{0}$ of 1 dB gap at BER $10^{-3}$. The differences in Figure 5 and Figure 6 also reflect the effect of certain key pilots for the channel estimation and equalization performance.

**Figure 7:**
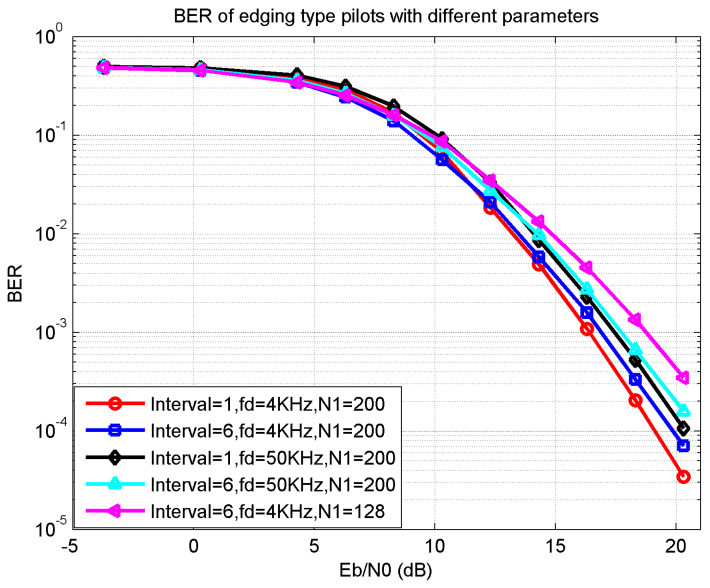
BER of edging type pilots with different parameters.

In Figure 7, the BER performance of the edging type pilots was simulated with different parameters. The initial interval between two adjacent pilots determines the initial pilot number; the smaller the interval the more pilots are inserted and the better the BER performance the system achieves to restore the channel impulse response. The occupied frequency bins, $N_{1}$, represents the possibility of more pilots to some extent. Therefore, the smaller number of $N_{1}$ the worse the BER performance. Compared with $N_{1}=200$ case, the $N_{1}=128$ case was much worse. The Doppler frequency $f_{d}$ increased the difficulty of channel estimation; a larger Doppler frequency leads to a worse BER performance.

**Figure 8:**
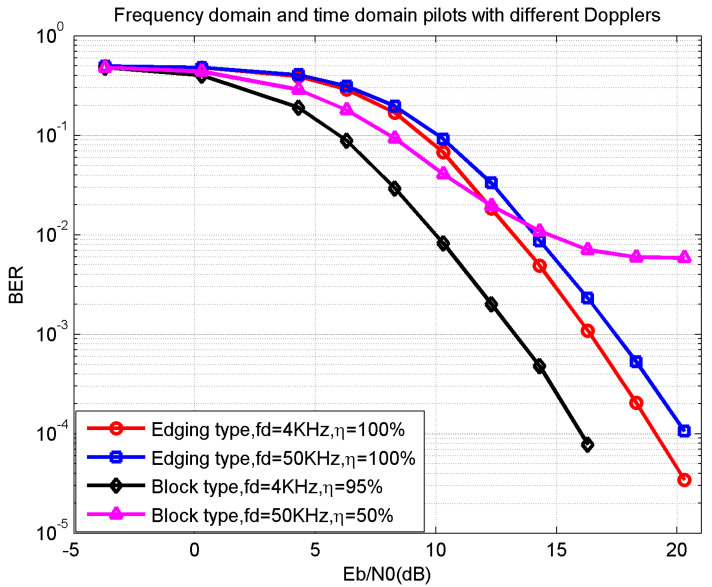
BER and efficiency between frequency domain and time domain pilots with different Dopplers.

In Figure 8, a traditional block-type pilot in a small Doppler of $f_{d}=4\;\mathrm{KHz}$ (slow-varying channel) condition showed the best BER performance, with an SNR $E_{b}/N_{0}$ 2.5 dB lower than the proposed method at BER $10^{-3}$. However, according to the definition of efficiency in (18) and (20), it also had the lowest efficiency η=50%. The platform of the triangle-line represents that the existing block-type pilot could not achieve in the fast-varying channels. As a comparison, the proposed edging-type pilot achieved an efficiency of η=100% with a much larger Doppler of $f_{d}=50\;\mathrm{KHz}$. The SNR $E_{b}/N_{0}$ gap was about 6 dB lower than the existing block-type pilot. Therefore, the proposed method had a much higher efficiency and better BER performance in the fast-varying channels.

**Figure 9:**
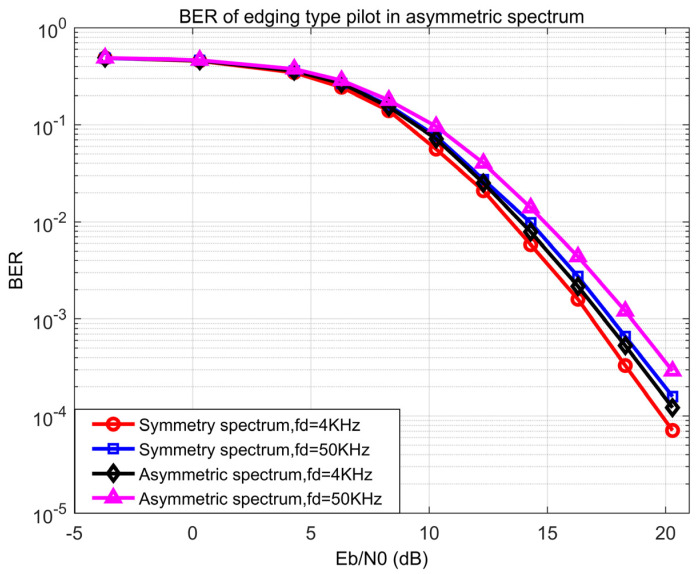
BER of edging type pilot in symmetric and asymmetric spectra.

To verify the availability of the proposed method in the asymmetric spectrum conditions, the BER performance of the edging-type pilot was simulated in symmetric and asymmetric spectrum conditions with different Dopplers. Based on the spectrum condition of the transmitter side, the spectrum was randomly changed for 5 to 10% of the whole bandwidth to simulate the typical asymmetry on the receiver side. Figure 9 shows that the proposed method in the asymmetric spectrum condition was about 1 dB better than the symmetric condition at BER $10^{-3}$ with a Doppler of 50 KHz and 0.5 dB with a Doppler of 4 KHz. The result represents that the proposed method is valid on the asymmetric spectrum conditions with a large Doppler.

## 5. Conclusions and Outlooks

This study presents an efficient frequency domain non-uniform pilot design method for TDCS, to enhance the adaptation of IoT cognitive radio devices in the non-continuous and asymmetric spectrum conditions. Based on the idea of the comb-type pilot in OFDM, considering the actual non-continuous and asymmetric spectrum, three frequency domain non-uniform pilots were proposed as the intersecting, the skewing, and the edging type. Then, the corresponding system estimation and equalization flow was presented with performance analysis. Some numerical examples were also presented with multipath model COST207RAx4 to verify the proposed methods in pilot distribution, BER and MSE. The simulation showed that the proposed methods achieved a much better channel estimation as well as efficiency performance than the existing block-type pilot method. The edging-type pilot had an obvious performance advantage over the others with a few extra pilot costs. The proposed method had a considerable performance in the large Doppler and asymmetric spectrum channel conditions

As a promising CR candidate, TDCS has a series of actual technical problems to solve, such as efficiency, environmental adaptation, peak-to-average power ratio (PAPR), etc. However, various aspects of capacity in TDCS are mutually restrictive. To put the proposed frequency domain non-uniform pilot into practical use, future work should consider the PAPR reduction for the inserted pilots.

## Data Availability

Not applicable.
